# Supplementary material for: Bond selectivity in electron-induced reaction due to directed recoil on an anisotropic substrate
Source: Nat Commun. 2016 Dec 9;7:13690. doi: 10.1038/ncomms13690 (PMC5476794; doi:10.1038/ncomms13690)
Supplement: Supplementary Information — Supplementary Figure 1 and Supplementary Note 1 [file ncomms13690-s1.pdf]

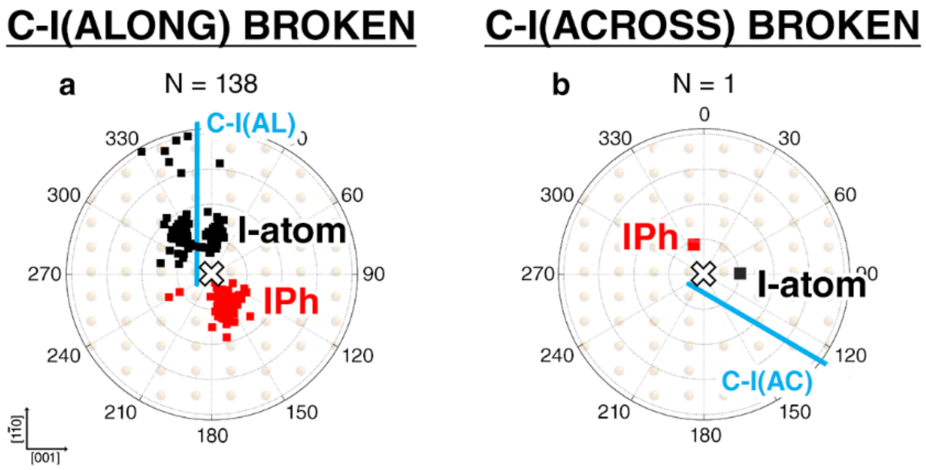

Supplementary Figure 1. Observed product distribution of the electron-induced reaction of mDIB on Cu(110). a, Polar plot for the breaking of C-I(AL). **b**, Polar plot for the breaking of C-I(AC). The reaction product recoil was measured relative to the intact reagent molecule (see white cross in Figure 1 of the main text). Because of the symmetry of the Cu(110) substrate, the data was folded with C-I(AL) along [1$\bar{1}$0], and C-I(AC) approximately along [001]. The positions of the I-atoms are given as black squares, and IPh (the brightest spot in STM image) as red squares. The distance between two adjacent concentric circles in the polar plots is 3.61 Å. The C-I bond direction in the physisorbed state responsible for the observed product scattering is given as a blue line in each case; (**a**) for Along and (**b**) for Across.

Supplementary Note 1

Details of reaction product distribution

Supplementary Figure 1 shows the observed spatial distribution in the reaction products relative to the prior physisorbed mDIB reagent (white cross in Figure 1 of the main text). The I-atom and IPh products were found to either side of the reagent as expected for these recoiling species. For the major path in which C-I(AL) bond broke, Supplementary Figure 1a shows that the I-atom had recoiled upward with respect to the reagent, whereas the IPh radical had recoiled downward. These opposite recoil directions of the two products of surface reaction are in accord with the model of electron-induced repulsion between them in a temporary anionic state, presented in the main text. The I-atom recoil was predominantly along the prior C-I(AL) bond-direction (blue line in Supplementary Figure 1a). For the minor path in which C-I(AC) bond broke, Supplementary Figure 1b, recoil was observed along C-I(AC) (blue line in Supplementary Figure 1b) with the I-atom recoiling to the right along C-I(AC), the IPh to the left.
